# EnGraft: a multicentre, open-label, randomised, two-arm, superiority study protocol to assess bioavailability and practicability of Envarsus® versus Advagraf™ in liver transplant recipients

**DOI:** 10.1186/s13063-023-07344-7

**Published:** 2023-05-11

**Authors:** D. S. Wöhl, B. James, M. Götz, F. Brennfleck, I. Holub-Hayles, I. Mutzbauer, S. Baccar, S. M. Brunner, E. K. Geissler, H. J. Schlitt, on behalf of the EnGraft Trial Group, Florian W. R. Vondran, Uta Herden, Jens Mittler, Ulf Peter Neumann, Silvio Nadalin, Andreas A. Schnitzbauer, Falk Rauchfuß, Felix Braun, Katharina Willuweit, Johann Pratschke, Thomas Berg, Thomas Vogel, Uta Merle, Roland Croner

**Affiliations:** 1 Department of Surgery, University Hospital Regensburg, Franz-Josef-Strauß-Allee 11, 93053 Regensburg, Germany; 2 coTrial Associates, Department of Surgery, University Hospital Regensburg, Franz-Josef-Strauß-Allee 11, 93053 Regensburg, Germany

**Keywords:** EnGraft, Envarsus, Liver transplantation, Superiority study, Randomised controlled trial

## Abstract

**Background:**

Graft rejection and chronic CNI toxicity remain obstacles to organ transplant success. Current formulations of tacrolimus, such as Prograf® and Advagraf™, exhibit limitations in terms of pharmacokinetics and tolerability, related in part to suboptimal bioavailability. As dosing non-compliance can result in graft rejection, the once daily formulation of tacrolimus, Advagraf™, was developed (vs 2x/day Prograf®). Benefits of Advagraf™ are counterbalanced by delayed achievement of therapeutic trough levels and need for up to 50% higher doses to maintain Prograf®-equivalent troughs. Envarsus® is also a prolonged-release once-daily tacrolimus formulation, developed using MeltDose™ drug-delivery technology to increase drug bioavailability; improved bioavailability results in low patient drug absorption variability and less pronounced peak-to-trough fluctuations. In phase III de novo kidney transplant studies, Envarsus® proved non-inferior to twice-daily tacrolimus; however, no phase IV studies show superiority of Envarsus® vs Advagraf™ in de novo liver transplant (LTx) recipients.

**Methods:**

The EnGraft compares bioavailability and tests superiority of Envarsus® (test arm) versus Advagraf™ (comparator arm) in de novo LTx recipients. A total of 268 patients from 15 German transplant centres will be randomised 1:1 within 14 days post-LTx. The primary endpoint is dose-normalised trough level (C/D ratio) measured 12 weeks after randomisation. Secondary endpoints include the number of dose adjustments, time to reach first defined trough level and incidence of graft rejections. Additionally, clinical and laboratory parameters will be assessed over a 3-year period.

**Discussion:**

C/D ratio is an estimate for tacrolimus bioavailability. Improving bioavailability and increasing C/D ratio using Envarsus could reduce renal dysfunction and other tacrolimus-related toxicities; previous trials have shown that a higher C/D ratio (i.e. slower tacrolimus metabolism) is not only associated with improved renal function but also linked to reduced neurotoxic side effects. A higher C/D ratio could improve clinical outcomes for LTx recipients; EnGraft has begun, with one third of patients recruited by January 2022.

**Trial registration:**

This trial has been registered (4 May 2020) in the EU Clinical Trials Register, EudraCT-Nummer: 2020–000796-20. Additionally, this trial has been registered (22 January 2021) at ClinicalTrials.gov: NCT04720326. The trial received a favourable opinion from the concerned lead ethics committee at the University of Regensburg, under the reference 20–1842-112.

## Background

Liver transplantation (LTx) is the treatment of choice for patients with end-stage liver disease. The liver is the second most frequently transplanted organ with a transplantation rate of 5500 livers per year in Europe [1].

Since the first experimental LTx in 1963, survival rates have improved significantly. This trend can be attributed to improved surgical techniques, donor organ preservation methods and, in particular, to the development and improvement of immunosuppressive medication [2]. The current 1-year post-transplant survival rates for patient and graft in LTx programs are approximately 85% and 80%, respectively; 5-year survival rates for most organ transplant programs range from 50 to 70% [1].

Despite major advances in transplantation medicine, graft rejection remains the key obstacle to long-term success. In clinical practice, a combination of two or three immunosuppressants is normally used to control the immune response in the immediate post-LTx period; this multi-drug regimen typically involves a combination of glucocorticoid (e.g. prednisone), calcineurin inhibitor (CNI) (cyclosporine or tacrolimus) and anti-proliferative agent (e.g. mycophenolate mofetil, azathioprine or everolimus). Other therapeutic agents include interleukin-2 receptor antagonists, such as basiliximab, often used as induction therapy to delay or reduce the need for CNIs. Once graft function has stabilised and the early immune response is controlled, the number and dose of immunosuppressive medications may be gradually reduced to minimise the wide range of overlapping adverse effects that include increased susceptibility to opportunistic infection and increased risk of malignancy.

Therefore, effective immunosuppressive strategies must strike a balance between suppressing the immune response against the transplanted organ whilst simultaneously minimising toxic side effects of the pharmacological interventions. Furthermore, side effects of the immunosuppressive therapy may affect patient compliance with the drug regimen, which is vital for maintaining immunosuppression and therefore assuring graft survival.

Tacrolimus, a macrolide CNI, was introduced to transplant practice in the early 1990s and since then has become the first-line immunosuppressive medication in most LTx programs [1, 3].

Currently marketed formulations of tacrolimus, such as Prograf® and Advagraf™, exhibit limitations primarily in terms of pharmacokinetic properties and tolerability. Tacrolimus has a narrow therapeutic index; blood trough level concentrations between 3 and 15 ng/ml are recommended during the first year after transplantation, provided that tacrolimus is given in combination with mycophenolate, steroids and induction with an anti-CD25 antibody [4]. Tacrolimus levels below this recommended range bear the risk of graft rejection, whereas levels above this range result in increased toxicity (e.g. nephrotoxicity, diabetes, tremor, hypertension) and increased susceptibility to opportunistic infections and malignancies [5].

Both Prograf® and Advagraf™ exhibit significant inter- and intra-individual variability in absorption owing to interactions with food and concomitant medications; also functional polymorphisms in the cytochrome P-450 system cause inconsistencies. In addition, both Prograf® and Advagraf™ exhibit a rather low bioavailability due to poor water solubility and pre-systemic gastrointestinal metabolism [6, 7]. As a result of this variability, standard dosing based on body weight is not an accurate predictor of drug exposure, and regular assessment of tacrolimus blood trough level concentrations is essential to measure effective drug exposure [8]. Currently, life-long monitoring of tacrolimus whole blood trough levels is required for all transplant patients [9].

Transplant recipients also typically require an array of concomitant medications for comorbidities, which means that interaction and tolerability profiles may cause issues. In addition, the intake schedule of many different medications may not easily be integrated into the patients’ daily life, which increases the risk of low patient compliance. As non-compliance with immunosuppressant dosing can result in graft rejection and failure, the once daily formulation of tacrolimus, Advagraf™, was designed to reduce the daily pill burden compared to Prograf® (taken twice daily). However, this potential benefit of Advagraf™ is counterbalanced by delayed achievement of therapeutic trough levels after transplantation and the need for an up to 50% higher dosage to maintain trough levels similar to Prograf® [10], therefore jeopardising tolerability.

A new once-daily formulation of tacrolimus, Envarsus®, was approved in 2014 by the European Medicines Agency (EMA) for the prophylaxis of rejection in kidney or liver allograft recipients and treatment of rejection resistant to other immunosuppressants [11]. Envarsus® is a prolonged-release formulation of tacrolimus developed using MeltDose™ drug-delivery technology, which increases the bioavailability of poorly water-soluble compounds via solid formulation at molecular state. Improvement in the bioavailability of Envarsus®, which allows once-daily administration, results in lower inter- and intra-patient variability of drug absorption with reduced peak-to-trough fluctuation and earlier achievement of a stable tacrolimus profile, as demonstrated in healthy volunteers [12]. In phase III studies using stable, de novo kidney transplant patients, Envarsus® has proven to be non-inferior to twice-daily tacrolimus in terms of treatment failure, a composite trial endpoint of death, graft failure, acute rejection or lost-to-follow-up [13, 14]. Additional potential benefits of Envarsus® are reduced pill burden and diminished peak-related toxicities such as tremor [15].

## Rationale

To characterise the metabolic profile of patients, several research groups have presented the calculation of the ratio between concentration in the blood and the drug dosage (C/D ratio) as a simple, practical and cost-neutral method easily applied during daily clinical practice, since trough level measurement is a necessary and routine part of therapeutic drug monitoring [16, 17]. A high C/D ratio indicates a slow rate of tacrolimus metabolism (i.e. lower doses of drug sufficient to maintain therapeutic blood concentrations); a low C/D ratio indicates a high rate of metabolism (i.e. higher doses of drug needed to maintain therapeutic exposure). Elevated tacrolimus peak levels are postulated as a possible reason for impaired kidney function and CNI nephrotoxicity in both liver and kidney transplant patients with low C/D ratio [16, 18]. The improved pharmacokinetic profile of Envarsus® with significantly lower peak levels in comparison to Advagraf™ [19] might be beneficial for these patients. It is widely reported that switching kidney transplant recipients to Envarsus® elevates the C/D ratio [20], since lower drug doses are sufficient to maintain therapeutic trough levels with this galenic drug formulation. Data from observational studies show that a higher C/D ratio can also be achieved when using this drug in LTx recipients [21, 22]. Since bioavailability studies on Envarsus® in the context of LTx are less prevalent in the scientific literature compared to kidney transplantation, and no phase III or IV studies with Envarsus® have been conducted in de novo LTx recipients, the EnGraft Study is designed to compare the bioavailability and practicability (handling) of two once-daily tacrolimus formulations Envarsus® versus Advagraf™ in de novo LTx recipients over 12 weeks. The recently identified and postulated parameter C/D ratio as an estimate for tacrolimus bioavailability will be calculated at different time points and will contribute to the pharmacokinetic profile of patients. The primary objective of EnGraft is to show that Envarsus® confers a superior (higher) C/D ratio in LTx recipients after 12 weeks of therapy under the conditions of a prospective, controlled, randomised clinical trial.

Several studies have identified a relationship between C/D ratio and clinical outcomes in organ transplant recipients. There is strong evidence that a lower C/D ratio (i.e. faster tacrolimus metabolism) is associated with inferior kidney function [23], a higher risk for CNI-induced nephrotoxicity [24] and greater susceptibility to BKV infection [25]. Conversely, studies in kidney transplantation have demonstrated that a higher C/D ratio (i.e. slower tacrolimus metabolism) is linked to a reduction in neurotoxic side effects such as tremor, headache and insomnia [20]. Moreover, a recently published observational study in LTx [21] has shown that a higher C/D ratio is associated with improved renal function, as measured by estimated glomerular filtration rate (eGFR).

Accordingly, it should be possible to limit renal dysfunction and reduce other tacrolimus-related toxicities by using a drug formulation that improves bioavailability and increases the C/D ratio. Therefore, a secondary aim of the EnGraft study is to prospectively test whether an elevated C/D ratio is associated with improved clinical outcomes. This is especially relevant in a patient population that is particularly vulnerable to kidney damage. The widespread use of the MELD (Model for End-Stage Liver Disease) score in determining organ allocation means that most LTx recipients already have impaired renal function at LTx. Since renal impairment is a typical side effect of CNI therapy, these patients are sensitive to CNI-induced nephrotoxicity very early after transplantation. The ReSpECT Study has already shown that lowering CNI exposure early after LTx is associated with better long-term kidney function [26]. Since C/D ratio correlates positively with eGFR after LTx [18], the EnGraft study aims to show that an elevated C/D ratio in the early post-transplant period can protect and preserve renal function without compromising liver allograft survival.

Since C/D ratio may be an effective tool for identifying patients at higher risk of developing poorer long-term outcomes in terms of kidney function and CNI toxicity, this study incorporates a long-term follow-up phase. By using C/D ratio to characterise patients in terms of tacrolimus metabolism at 12 weeks post-transplant, it will be possible to evaluate whether the sub-groups of slow, intermediate and high metabolisers exhibit long-term clinical outcomes that correlate with their metabolic profile. This would support the hypothesis that the C/D ratio has a predictive value that, when calculated during the early stages after LTx, may help transplant clinicians to identify patients at risk of long-term complications and to individualise patient therapy accordingly.

Therefore, the EnGraft Study will follow patients for 3 years in total in order to assess long-term efficacy and safety outcomes and to see whether these outcomes correlate with the metabolic profile measured during the early post-transplant period.

Controlled, prospective, multicentre trials are now needed to test the predictive value of the tacrolimus C/D ratio. Whilst published literature regarding C/D ratio in LTx is encouraging, we are currently reliant on retrospective, single-centre studies using small and heterogeneous patient cohorts. There are also very few direct pharmacokinetic comparisons of the two once-daily tacrolimus formulations (Advagraf™ and Envarsus®). The EnGraft study is the first large, multicentre, randomised, controlled study in LTx to test Envarsus® using C/D ratio as the primary variable.

## Study design

EnGraft is a prospective, randomised, controlled, multicentre, open-label, two-arm, parallel-group, phase IV clinical trial to assess bioavailability and practicability of *En*varsus® compared with Adva*graf*™ in de novo liver *t*ransplant recipients (EnGraft).

Two hundred and sixty eight (268) patients at 15 LTx centres in Germany will be enrolled and randomised in a 1:1 ratio to two study arms. Patients in the test arm will be treated with Envarsus® (test) as first-line CNI in the immunosuppressive regimen. The control arm will treat patients with Advagraf™ (comparator) as first-line CNI. Trial participants will additionally receive all of the other treatments that comprise a standard, multi-drug, immunosuppressive regimen for LTx recipients, according to routine clinical practice in Germany.

Since this trial compares two commercially available medications with different pharmaceutical formulations (Envarsus® tablets versus Advagraf™ capsules), simple, cost-neutral treatment blinding is not possible. The trial is being conducted as an open-label study.

LTx will be performed prior to study entry, according to the local standard at participating trial centres. An optional run-in phase is available, in case patients cannot be randomised immediately after transplantation surgery (e.g. unable to swallow study medication). Patients may be enrolled up to 14 days after surgery.

The day of randomisation to EnGraft is defined as trial day 0. After a 12-week controlled treatment phase, patients enter a follow-up phase of 2 years and 9 months. The final study visit is scheduled for 3 years after randomisation.

**Fig. 1:**
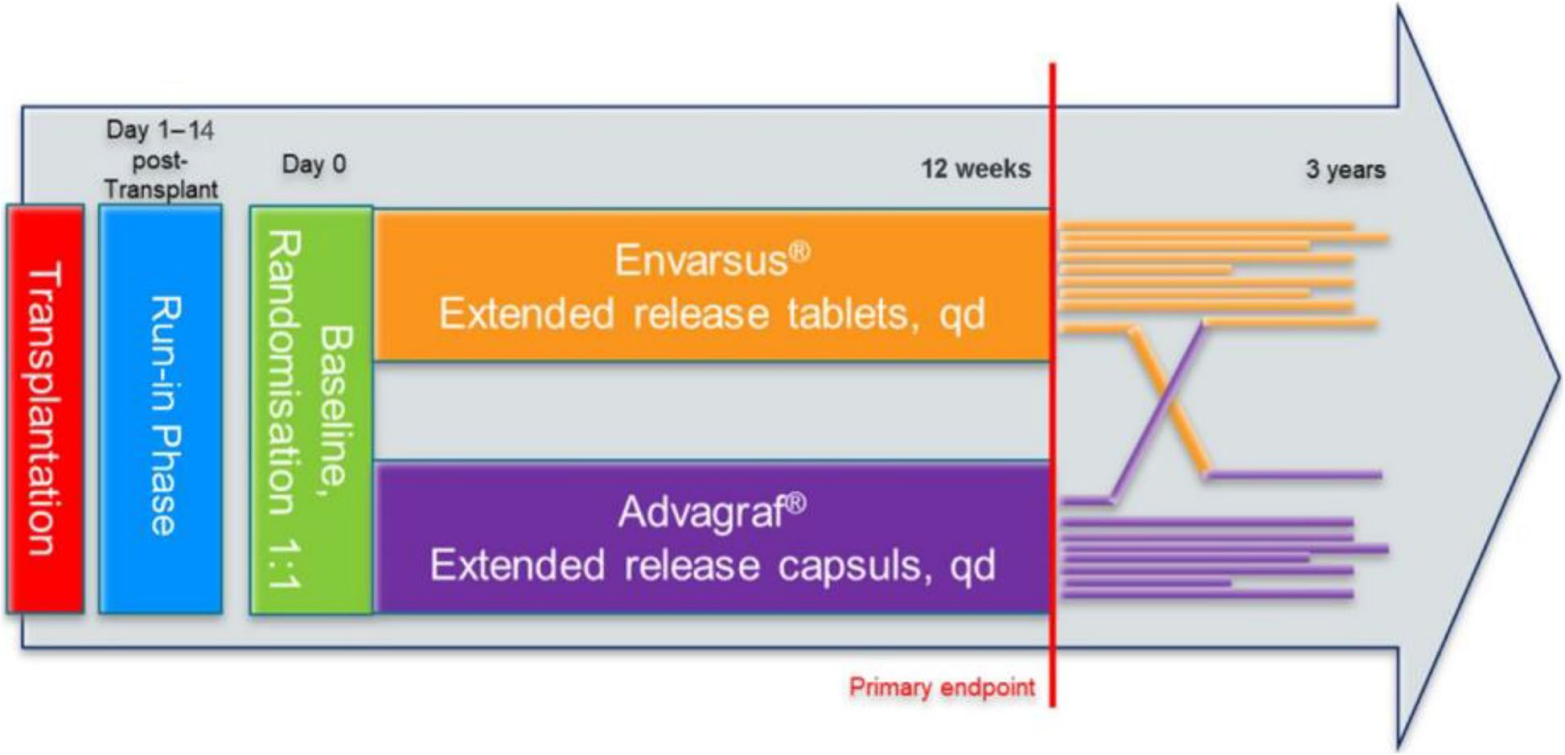
Study flowchart. Showing trial phases and treatment arms

The study design adheres to the principles outlined in ICH-GCP E6 (R2). All patients aged ≥ 18 years who meet the eligibility criteria and give informed consent will be allocated to the study. Those patients are supplied with patient information. For each trial participant, an authorised and delegated trial investigator must obtain written informed consent after sufficient time for reflection prior to conducting any trial-related procedures involving the subject. After consent is obtained, patients are randomised to the trial. The used patient information sheet and the informed consent are both ethically approved (Fig. 1).

## Objectives

### Primary endpoint

**Fig. 2:**
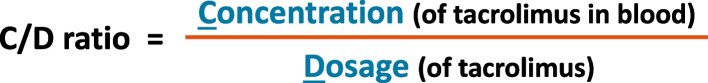
Calculation of C/D ratio. In this formula, “C” represents the trough level measured in a blood sample collected immediately prior to drug dosing on the day of a trial visit (*t*_0_) and “D” denotes the daily dose taken by the patient on the day prior to the visit (*t*_-1_)

All time points for study endpoints are measured relative to patient randomisation (day 0). The primary variable is the C/D ratio measured at 12 weeks. C/D ratio is being measured in this study as a surrogate for tacrolimus bioavailability (i.e. systemic exposure per mg of drug) and will be calculated according to the formula in Fig. 2.

A centralised measurement of the 12-week tacrolimus blood trough level at the University Hospital Regensburg will be used to calculate C/D ratio for the primary endpoint.

**Fig. 3:**
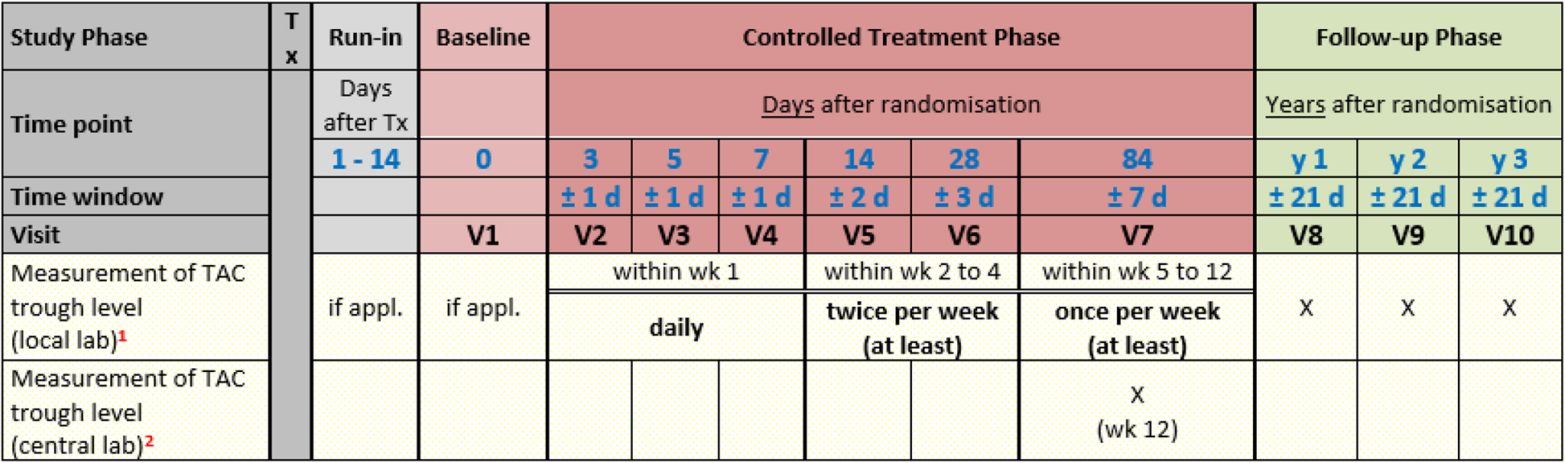
Tac measurements. Minimum schedule of tacrolimus trough level measurements

During the controlled treatment phase (first 12 weeks post-randomisation), tacrolimus should be dosed by the investigator to reach and maintain whole blood trough concentrations within the general reference range of 3–12 ng/ml. For every randomised patient, a narrower target range (with an interval of 3 ng/ml) within the wider reference range of 3–12 ng/ml is to be defined at all times and continually reviewed/revised based on clinical circumstances. The first target range is to be set prospectively by the investigator and documented in the eCRF at baseline to enable analysis of the pharmacokinetic endpoints. Since the frequency of trough level measurement directly influences pharmacokinetic endpoints, each trial centre aims to follow the minimum schedule of trough level measurement outlined in Fig. 3.

C/D ratio tends to fluctuate during the first weeks and months after transplantation. Twelve weeks post-randomisation (visit 7) has therefore been chosen for timing of the primary endpoint because the overall metabolic situation in patients is expected to be stable by this time. Also, at 12 weeks, the influence of concomitant immunosuppressive agents that may affect C/D ratio should be less pronounced. Corticosteroids are often concomitantly administered with tacrolimus and are allowed as non-investigational medication products (NIMP) in the EnGraft study. Since they share some common metabolic and transporter pathways with tacrolimus, higher steroid doses and rapid steroid tapering during the early post-transplant period may interfere with tacrolimus metabolism and destabilise the C/D ratio [18, 27]. At the 12-week time point, the use of steroids should have been tapered and the C/D ratio sufficiently stable to provide an accurate estimate of tacrolimus bioavailability.

### Selected secondary endpoints

- Number of IMP dose adjustments until 12 weeks
- Time (days) to reach the first defined range in target trough level, utilising the date of first in-range reading of two consecutive readings within the range
- Number of measurements above and below the first defined range in target trough level
- C/D ratio measured at 1, 2 and 3 years
- Mean tacrolimus trough level and inter-patient variability (range) of tacrolimus trough levels at 1, 2, 4 and 12 weeks
- Inter-patient variability (range) of tacrolimus total daily dose until 12 weeks
- Proportion of patients with trough levels lower, within, or higher than the standard reference range at 1, 2, 4 and 12 weeks
- Incidence and severity (BANFF criteria) of clinically confirmed biopsy-proven rejection (BPAR) at 12 weeks and 1, 2, 3 years
- Incidence of graft failure (defined as necessity for re-transplantation) at 12 weeks and 1, 2, 3 years
- Incidence of death (for any reason) at 12 weeks and 1, 2, 3 years
- Laboratory measures of liver function, renal function and metabolism at 12 weeks and 1, 2, 3 years
- Malignancies and infections at 1, 2 and 3 years
- Incidence of de novo occurrence of tremor or vision impairments
- Incidence of post-transplant diabetes mellitus and post-transplant hyperglycaemia at 12 weeks and 1, 2, 3 years

Graft biopsies are read at each site by the local pathologist. The local assessment will be used to guide the clinical treatment of the patient during the study. A diagnosis of BPAR is based on histological grading using the BANFF criteria for hepatic allograft pathology. In addition, a central assessment of biopsy pathology will be conducted by an independent central pathologist who is blind to the treatment assignment. To avoid detection bias, the blinded central assessment will be taken as the official result for the trial and for the determination of the BPAR endpoint.

### Inclusion criteria

Patients must meet all of the following inclusion criteria to be eligible for randomisation:

- Signed and dated written informed consent
- Adult (≥ 18 years old) male or female
- Recipient of a whole liver transplant from a deceased donor or a split liver transplant from a deceased or living donor
- ABO blood type compatible with the organ donor
- Able to swallow an oral formulation of tacrolimus in tablet or capsule form

### Exclusion criteria

- Multi-organ transplantation
- Any previous organ allograft transplantation
- Biopsy-proven acute rejection that is ongoing at the time of randomisation
- Occurrence of post-transplant thrombosis, occlusion or stent placement in any major hepatic arteries, hepatic veins, portal vein or inferior vena cava
- History of extra-hepatic malignancy that could not be curatively treated
- Hepatocellular carcinoma with extra-hepatic spread or macrovascular invasion
- Uncontrolled systemic infection
- Requirement of life support measures such as ventilation or vasopressor agents (> 20 μg/kg BW/h) at the time of randomisation
- Known contraindication or hypersensitivity to tacrolimus, and/or to any of the excipients listed in Sect. 6.1 of the Summary of Product Characteristics (SmPC) of both Envarsus® and Advagraf™, and/or to any other macrolides
- Ongoing, planned or foreseeable use of cyclosporine or any tacrolimus preparation other than Envarsus® or Advagraf.™ (except for immediate-release formulations administered before randomisation)
- Any prolonged-release tacrolimus treatment prior to randomisation
- Pregnant or nursing (lactating) female, where pregnancy is defined as the state of a female after conception and until the termination of gestation, confirmed by a positive hCG laboratory test
- Female of child-bearing potential, defined as physiologically capable of becoming pregnant, unless using a reliable method of contraception
- Participation in another interventional clinical trial during the time period from randomisation to study end, if the trial is testing an IMP (AMG study) or if the intervention and/or follow-up requirements of the trial impede or interfere with either the objectives of EnGraft or the treatment/follow-up requirements of EnGraft
- Any condition or factor which, in the judgement of the investigator, would place the subject at undue risk, invalidate communication with the investigator or study team, or hamper compliance with the trial protocol or follow-up schedule
- Inability to freely give informed consent (e.g. individuals under legal guardianship)

All women of childbearing potential will undergo a serum pregnancy test prior to transplantation as per site routine. Postmenopausal women (physiologic menopause defined as 12 consecutive months of amenorrhea) or women who are permanently sterilised (e.g. tubal occlusion, hysterectomy or bilateral salpingectomy) may be enrolled in the study without serum pregnancy testing.

## Interventions

### Randomisation

A dynamic allocation technique will be used for randomisation of patients fulfilling the trial eligibility criteria in a 1:1 ratio to one of two treatment arms: Envarsus® tablets (test IMP) or Advagraf™ capsules (comparator IMP). Pre-treatment with immediate-release tacrolimus (Yes/No), as well as trial site, will be used as stratification factors in the treatment allocation to minimise sources of treatment bias. The first stratification factor (study centre) will minimise systematic treatment bias at the study centre level and reduce the influence of inter-centre variability. The second stratification factor (pre-treatment with immediate-release tacrolimus) will minimise treatment bias at the level of the individual patient, since patients treated with Prograf® during the run-in phase can be expected to develop stable trough levels after randomisation more rapidly than patients who do not receive Prograf® prior to commencing IMP. Balancing the treatment arms with respect to this factor allows fair and unbiased evaluation of the secondary pharmacokinetic endpoints that evaluate the ease with which the first target trough level is achieved.

The allocation algorithm is programmed into a randomisation module in the trial database. Study investigators at the participating trial sites have access to the randomisation module via the online trial database and randomise using this central tool. It is vital that randomisation is performed as soon as possible after eligibility has been confirmed and no later than 14 days after transplantation surgery; enrolment after this time period is not allowed.

### Investigational group

Envarsus® will be supplied by the sponsor for the duration of the 12-week controlled treatment phase. Envarsus® will not be supplied for the follow-up phase (Table 1).

**Table 1:** IMP-characteristics of Envarsus®

IMP name	Envarsus®
Active ingredient	Tacrolimus monohydrate
Pharmaceutical form	Prolonged-release tablet
Presentation	Oval, white to off-white uncoated tablet
Administration	Once daily, oral formulation provided in 0.75 mg, 1.0 mg and 4.0 mg dosage strengths

Envarsus® therapy should commence at a starting dose of 0.11–0.13 mg/kg/day.

In case the patient initially received an oral formulation of Prograf® during the run-in phase (e.g. administered via nasogastric tubing), conversion should be performed on a 1:0.7 (mg to mg) total daily dose basis. In case the patient initially received intravenous Prograf® therapy (continuous 24-h infusion) during the run-in phase at a dose approximately 1/5th of the recommended oral dose, the conversion to Envarsus® should be modified accordingly.

### Control group

Advagraf™ will not be provided by the sponsor, but supplied/prescribed as per site routine (Table 2).

**Table 2:** IMP-characteristics of Advagraf™

IMP name	Advagraf™
Active ingredient	Tacrolimus monohydrate
Pharmaceutical form	Prolonged-release hard capsule
Presentation	Gelatin capsule with light yellow/white/orange/greyish-red capsule cap on an orange capsule body containing white powder in respectively 0.5 mg/1 mg/3 mg/5 mg dosage strengths
Administration	Once daily, oral formulation provided in 0.5 mg, 1.0 mg, 3.0 mg and 5.0 mg dosage strengths

Advagraf™ therapy should commence at a starting dose of 0.1–0.2 mg/kg/day.

In case the patient initially received an oral formulation of Prograf® during the run-in phase (e.g. administered via nasogastric tubing), conversion should be performed on a 1:1 (mg to mg) total daily dose basis. In case the patient initially received intravenous Prograf® therapy (continuous 24-h infusion) during the run-in phase at a dose approximately 1/5th of the recommended oral dose, the conversion to Advagraf™ should be modified accordingly.

### Non-investigational medical products

In this trial, it is medically necessary in most cases for clinical trial subjects to receive a multi-drug immunosuppressive regimen to prevent allograft rejection. Therefore, in addition to IMP, patients will be treated with a therapy of several other immunosuppressive agents, regardless of randomisation group. All systemic immunosuppressive therapy given will be collected in the eCRF.

The following substances are prohibited during study participation:

- Cyclosporine
  - Any tacrolimus preparation other than the assigned IMP (excluding immediate-release formulations taken prior to randomisation or during an acute rejection episode or in place of the IMP during a temporary interruption of IMP)
  - St. John’s wort (*Hypericum perforatum*)
  - Grapefruit and grapefruit juice

## Data collection methods

### Run-in phase (optional)

The optional run-in phase starts immediately after organ transplantation surgery. During this phase, LTx recipients can be screened for inclusion in the EnGraft Study and may receive immunosuppression and concomitant medication as per local standard practice.

The following procedures will take place:

- Signed informed consent
- Evaluation of patient eligibility including, if applicable, pregnancy test
- Measurement of tacrolimus trough levels (if patient treated with an oral formulation of immediate-release tacrolimus)
- Testing for donor-specific antibodies (if per local practice)

### Visit 1—Baseline

At the baseline visit (visit 1), patients are randomised and baseline characteristics are measured. The date of the baseline visit is designated as trial “day 0” and must occur no later than 14 days after transplantation surgery.

In case the run-in phase is skipped, all screening procedures must be performed at the baseline visit prior to randomisation. Randomisation may take place only after all eligibility criteria have been checked and fulfilled.

**Fig. 4:**
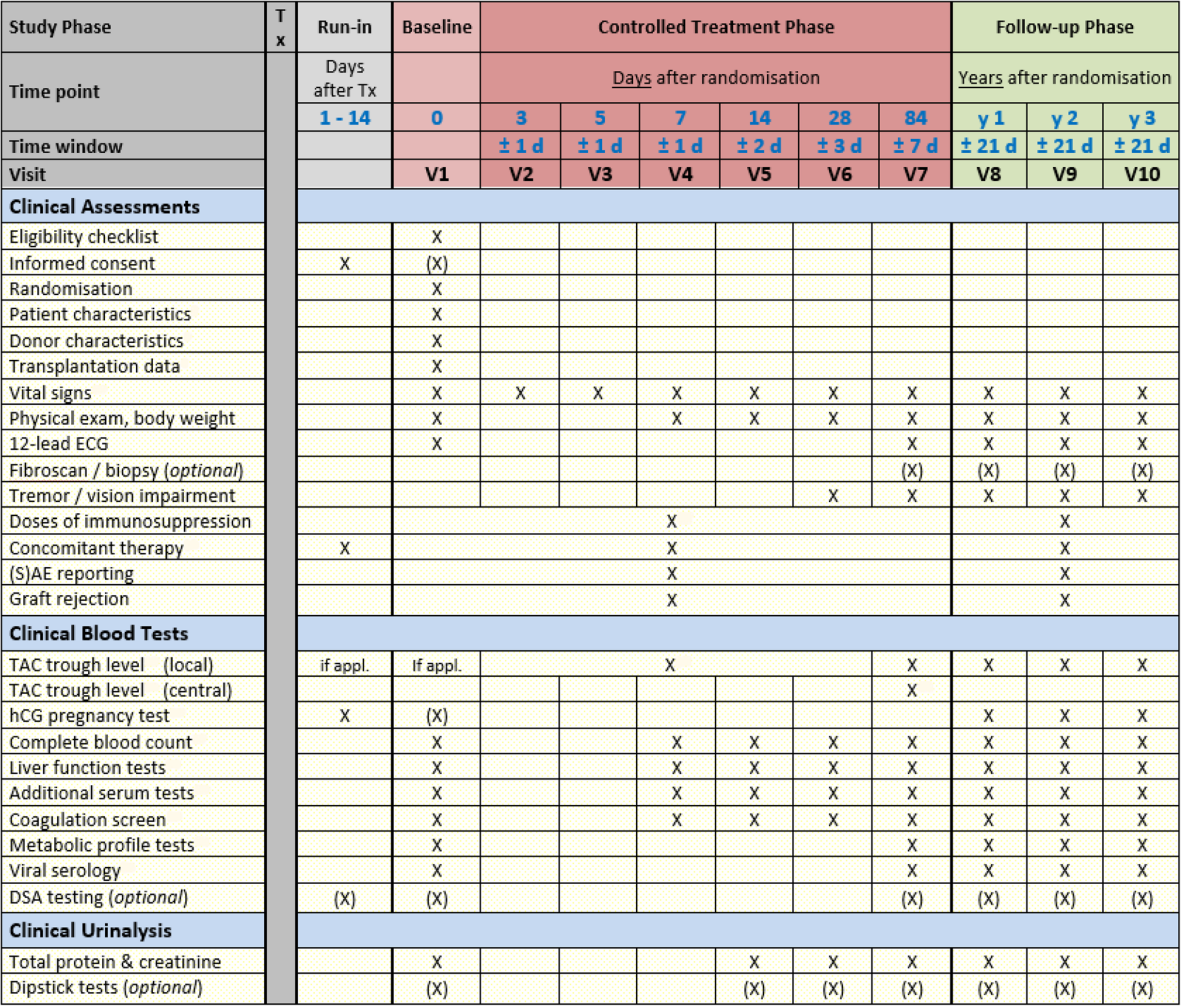
Schedule of clinical assessments

The following baseline procedures/data will be performed/collected (the applicable panel of assessments is displayed in Fig. 4):

- Randomisation, dispensing of assigned IMP, explaining practicalities of drug administration to the patient
- Patient characteristics, including demographics
- Past medical history
- Donor and transplantation data
- Physical examination, including body weight
- 12-lead ECG
- Vital signs: blood pressure, pulse rate, body temperature
- Blood sampling for clinical tests:
  - ◦ Haematology, serum biochemistry, including calculation of eGFR
  - ◦ Blood coagulation screen
  - ◦ Virology status: CMV, EBV, HCV, HBV
  - ◦ Tacrolimus trough level (if immediate-release tacrolimus given prior to randomisation)
- Urine sampling for clinical urine analysis
- Review and documentation of concomitant medication that interacts with IMP or is prohibited per protocol

### Visit 2 to visit 6 (day 3 to day 28) of controlled treatment phase

During the first 4 weeks after randomisation, five study visits are performed and assessments should be conducted according to Fig. 4. Tacrolimus trough level measurements should be performed (at least) according to the minimum scheduled outlined in Fig. 3. A time window of ± 1 day is permitted for visits 2–4, this time window extends to ± 2 and ± 3 days for visit 5 and visit 6, respectively.

At visit 6, the patient should be assessed for signs of de novo occurrence of tremor or vision impairments. If detected, these should be documented as adverse events.

### Visit 7 (day 84 ± 7 days) of controlled treatment phase

The applicable panel of assessments at visit 7 is displayed in Fig. 4, including an optional donor-specific antibody (DSA) test and an optional fibroscan/protocol biopsy. Visit 7 marks the 12-week time point that will be used to evaluate the primary endpoint (tacrolimus C/D ratio), which will be calculated using the trough level measured in the blood sample collected on the day of visit 7 (12 weeks) and the dose of study drug taken by the patient one day prior to visit 7. To standardise measurement of the visit 7 trough level across all participating study sites, an additional aliquot of EDTA-blood will be collected and shipped to the University Hospital Regensburg for central analysis.

### Visit 8 to visit 10 (year 1 to year 3 ± 21 days) of follow-up phase

The follow-up visits will take place annually until year 3, i.e. visit 8 (1 year after randomisation), visit 9 (2 years after randomisation) and visit 10 (3 years after randomisation).

The panel of assessments for the follow-up visits is shown in Fig. 4.

### Sample size calculation

Sample size estimation is based on testing superiority of Envarsus® versus Advagraf™ with respect to the primary efficacy variable. C/D ratio data obtained from previously published clinical trials in kidney transplantation and observational studies in LTx inform an assumed difference between the two treatments Envarsus® or Advagraf™ after 12 weeks of approximately 0.4 with a standard deviation of 1.0.

A sample size of 100 patients per treatment group will have the power of 80% to show a difference of 0.4 in C/D ratio between the two treatment groups with a two-sided significance level of 5%. The primary test will be performed based on the Full Analysis Set (FAS) but will be repeated for the Per-protocol analysis set (PPAS). To have a sufficient power also for the PPAS comparison, it is planned to randomise 134 patients in each treatment group assuming that about 25% of randomised patients cannot be included in the PPAS because of major protocol deviations. A sample size of 134 patients per treatment group will have a 90% power to show a difference of 0.4 in C/D ratio in the FAS. The minimal detectable difference in the FAS will be 0.24.

### Hypotheses

The following null (H_0_) and alternative (H_a_) hypotheses will be tested for the primary endpoint (C/D ratio) at a two-sided significance level of α = 0.05:

$${H}_{0}: {\mathrm{Mean}}_{\mathrm{Envarsus}}= {\mathrm{Mean}}_{\mathrm{Advagraf}} , \mathrm{versus}$$

$${H}_{\mathrm{a}}: {\mathrm{Mean}}_{\mathrm{Envarsus}} \ne {\mathrm{Mean}}_{\mathrm{Advagraf}}$$

in which Mean _Envarsus_ and Mean _Advagraf_ refer to mean C/D ratio at visit 7 (12 weeks after randomisation) in the Envarsus® and Advagraf™ treatment groups, respectively.

### Population for analysis

The primary analysis will be based on the FAS. However, a sensitivity analysis will be done on the PPAS. All safety data will be analysed by means of the safety population.

### Full Analysis Set (FAS)

This consists of all randomised trial patients with at least one tacrolimus blood trough level reading after randomisation.

### Per-protocol analysis set (PPAS)

This consists of the FAS without any major protocol deviations (e.g. violation of eligibility, non-/poor compliance, non-permitted medications). The precise definition of the PPAS will be specified by the sponsor after review of aggregated data without information on individual treatment assignment, prior to database lock for the primary analysis.

### Safety population

This consists of all trial patients who receive at least one dose of study IMP after randomisation. The safety population will be used for analysis of all safety variables.

### Statistical analysis

The statistical analysis will be done by Excelya Germany GmbH. All statistical analyses will be carried out using SAS® 9.4 or higher.

For efficacy and PK endpoints, all patients will be analysed as belonging to their randomised IMP, regardless whether patients received a treatment different from the treatment to which they were randomised. For safety endpoints, all patients will be analysed according to the treatment they actually received.

### Patient demographics and baseline characteristics

Demographics and baseline characteristics/assessments will be summarised in total and by treatment arm using descriptive statistics.

The following variables will be summarised: age, gender, race, BMI, medical history, physical examination, vital signs, 12-lead ECG, laboratory data and donor characteristics.

### Primary endpoint

The aim of the EnGraft Study is to show that Envarsus® is superior to Advagraf™ with respect to the primary variable, which is defined as the C/D ratio at week 12 after randomisation.

The analysis of the primary endpoint will be performed by means of analysis of covariance considering randomised treatment and pre-treatment with immediate-release tacrolimus (stratification factor) as fixed effects in the model. Least square means, standard errors, treatment difference and corresponding 95% confidence intervals will be reported.

The primary population for analysis of the primary endpoint, secondary efficacy endpoints and secondary pharmacokinetic endpoints will be the FAS. Supportive analyses will be carried out for the PPAS.

### Secondary endpoints

All secondary variables will be analysed by treatment group using descriptive statistical methods. Secondary efficacy variables for the controlled treatment phase may be analysed between the two groups using two-sided testing with nominal significance level *α* = 0.05 and are to be interpreted in a strictly exploratory sense. If appropriate, two-sided 95% confidence intervals will be provided.

### Safety endpoints

The safety analysis will be performed using the safety population. All safety data will be listed for the two treatment arms.

### Missing data

A multiple imputation approach will be used to account for missing values of the primary variable at week 12.

### Interim analysis

No interim analysis is planned for this trial.

### Cross over therapy/non-compliance

Patients should not be switched to any other formulation of tacrolimus unless it is for a justified medical reason. In case of interruption or switching of tacrolimus therapy, patients should be returned to the assigned IMP as soon as it is safe to do so. Non-compliance with IMP intake is possible, especially after patients are discharged from the hospital. To optimise patients’ compliance, a compliance check will be done at each site visit, including drug accountability and tacrolimus blood trough level measurements.

### Monitoring

Monitoring of the trial is performed by coTrial Associates (www.cotrialassociates.com), which is located within our Department of Surgery. Monitoring is carried out in accordance with standard operating procedures, using a risk-based approach. Regular on-site monitoring visits are performed. Investigators must allow the monitor to look at all source data and essential documents, support the monitor during visits and answer queries. All monitoring procedures and the extent of Source Data Verification (SDV) are predefined in a trial-specific monitoring manual.

### Safety

Since EnGraft is a phase-IV clinical trial using marketed IMPs within their licensed indication (LTx), there is no additional risk expected for randomised patients.

All adverse events and reactions will be documented in the eCRF and will be classified as serious or non-serious and IMP-related or not IMP-related by the investigator. All adverse events (AE) must be recorded in the eCRF database as soon as possible, but within 10 days of awareness. Serious adverse events (SAE) must additionally be reported on a SAE report form to the sponsor within 24 h of awareness. In the case that a SAE is classified as a SUSAR, the sponsor will report all relevant information concerning this SUSAR to the national competent authority, lead ethics committee, chief investigator and principal investigators at all participating trial centres within 15 days after sponsor awareness, in case of fatal or life-threatening SUSARs within 7 days after sponsor awareness. The report should contain date of onset of the event, outcome, date and cause of death (for fatal outcome), dosing of IMPs at the time of the event and the assessment of the causal relationship to all IMPs.

## Discussion

Tacrolimus is an effective immunosuppressive drug that is used immediately and long-term in the majority of LTx recipients. Currently available tacrolimus formulations are characterised by high inter- and intra-variability, high peak levels and low bioavailability, often creating problems in clinical efficacy. Envarsus® is manufactured using MeltDose technology and represents an innovation in the field of immunosuppressive drugs. This galenic formulation increases the amount of active ingredient that reaches the blood, ensuring higher therapeutic efficacy. In a phase 2 study conducted on stable LTx patients [28], pharmacokinetic data demonstrated consistent exposure at a lower conversion dose. In 2019, Baccarani et al. [22] retrospectively compared Envarsus® versus Advagraf™ in de novo LTx recipients, focusing on administered daily dose and therapeutic trough levels during the first 30 days after transplant. Using Envarsus® resulted in faster achievement of therapeutic trough levels. Moreover, after stabilisation of tacrolimus blood levels, patients given Envarsus® required a 25% lower median dose of administered drug to maintain the same therapeutic trough level compared with Advagraf™. Furthermore, Grinyó et al. [29] showed in 2014 approximately 30% lower dosing benefit using Envarsus® compared to twice-daily tacrolimus capsules (e.g. Prograf®). Tacrolimus levels below the recommended range bear the risk of graft rejection, whereas levels above this range result in increased toxicity (e.g. nephrotoxicity, diabetes, tremors, hypertension) and increased susceptibility to opportunistic infections and malignancies [5]. Using the MeltDose technology, Envarsus® shows a lower intra-day fluctuation in tacrolimus blood concentration compared to Advagraf™ [19]. Therefore, potential benefits of Envarsus® are reduced pill burden and diminished peak-related toxicities such as tremors [15]. Thus, these factors will be measured and quantified by the secondary endpoints we have chosen.

The recently identified C/D ratio as a cost-effective estimate for tacrolimus bioavailability will be calculated at 12 weeks after randomisation as the primary endpoint of EnGraft. Bioavailability studies on Envarsus® in the context of LTx are less prevalent in the scientific literature compared to kidney transplantation, and no prospective phase III or IV studies with Envarsus® have been conducted in de novo LTx recipients. Our aim is to show that Envarsus® confers a superior (higher) C/D ratio in LTx recipients after 12 weeks of therapy. Several studies have identified a relationship between C/D ratio and clinical outcomes in organ transplant recipients. Sánchez Fructuoso et al. demonstrated that a higher C/D ratio is linked to a reduction in neurotoxic side effects, e.g. tremor, after kidney transplantation [20]. Moreover, in 2020 von Einsiedel et al. showed in their observational study after LTx that a higher C/D ratio is associated with improved renal function, as measured by eGFR [21]. If EnGraft is able to confirm these findings and to show superiority of Envarsus® over Advagraf™ in terms of C/D ratio as hypothesised, a higher C/D ratio could improve clinical outcomes for LTx recipients and confer a real clinical benefit for Envarsus®-treated patients that will be detected by our chosen secondary endpoints.

EnGraft consists of an optional run-in phase which may last for up to 14 days after transplantation surgery. Within this period, patients may be pre-treated with an immediate-release formulation of tacrolimus (e.g. Prograf®). Tacrolimus target trough levels may be reached faster in this pre-treated subset of patients and unfairly bias the secondary endpoints that are based on drug pharmacokinetics. For this reason, “pre-treatment” was chosen as a stratification factor for the dynamic randomisation algorithm to enable a fair and unbiased evaluation of the secondary pharmacokinetic endpoints. Additionally, patients spending different amounts of time in the run-in phase prior to randomisation consequently affects the time when patients are discharged from the transplantation centres relative to the study visits. This factor may influence compliance with the minimum scheduled trough level measurements resulting in protocol deviations and potentially influencing our chosen secondary endpoints.

Results in trough level measurement are only interchangeable if analytic methods are the same. For therapeutic drug monitoring, especially tacrolimus trough level measurement, different types of immunoassays and the tandem mass spectrometry are used depending on local laboratory standards [30]. According to the results of the UKNEQAS proficiency testing, these different methods show different sensitivities, thus resulting in variance in tacrolimus trough level readings. Especially for evaluation of the primary endpoint, it is necessary that comparable tacrolimus trough levels are obtained to reduce this systemic bias. For this reason, a centralised trough level measurement at the University Hospital Regensburg will be used to calculate C/D ratio for the primary endpoint at V7 (12 weeks).

## Trial status

The currently valid version of the protocol is V2.0_2020-07–31. The recruitment phase started on December 14, 2020, until estimated June 2023.

## Abbreviations

AE: Adverse event
DSA: Donor-specific antibodies
BMI: Body mass index
BKV: BK-virus
BPAR: Biopsy-proven acute rejection
CMV: Cytomegalovirus
CNI: Calcineurin inhibitor
EBV: Epstein-Barr virus
ECG: Electrocardiogram
eCRF: Electronic case report form
eGFR: Estimated glomerular filtration rate
EMA: European Medicines Agency
EU: European Union
FAS: Full Analysis Set
HBV: Hepatitis-B virus
hCG: Human chorionic gonadotropin
HCV: Hepatitis-C virus
ICH: International Conference on Harmonisation
LTx: Liver transplantation
(N)IMP: (Non-)Investigational Medicinal Product
MELD: Model for End-Stage Liver Disease
PPAS: Per-protocol analysis set
SAE: Serious adverse event
SDV: Source Data Verification
SmPC: Summary of Product Characteristics
SUSAR: Suspected Unexpected Serious Adverse Reaction
Tac: Tacrolimus
